# Dr. Edward Maynard

**Published:** 1891-06

**Authors:** 


### DR. EDWARD MAYNARD.

The death of Dr. Maynard, noticed in full in another part of this journal, is an event in dentistry. The present generation of workers, it is to be presumed, have only a traditional idea of his labor and of the position he once occupied. To those, however, who were contemporary with him, or as young men of the past who reverently spoke his name, his death recalls one of the most interesting periods in the building up of the profession.

To Dr. Maynard belongs the credit of dividing the old dentistry from the present, in fact it may be said that he made the dentistry of to-day possible by the introduction of his mode of filling root-canals. This he did, and so thoroughly and so skilfully was this performed that the results carried conviction to the minds of the best workers everywhere.

When it is remembered that prior to this the salvation of teeth with exposed pulps was an impossibility save in exceptional cases, that little was known of the pathological complications liable to ensue, it is not surprising that Dr. Maynard's work opened up possibilities more important in their far-reaching effects than anything that followed, not even excepting the introduction of cohesive gold. It has long been the teaching of the writer that the operation of canal-fillings was the beginning of modern dentistry, that all that preceded it was in degree empirical, and that all that has since followed has more and more approached a truly scientific basis.

Dr. Maynard was a many-sided man. He was in a measure lost to dentistry by other and, to him, more important work, but the fact that he did so much should cause his name to be honored as one who made dentistry worthy its present position.

Dr. Maynard may have built better than he knew. Before the period of antisepsis so effectually did he do his work that to-day we cannot do any better, and in the skilful handling of material for filling canals there is nothing done at present as Maynard, Townsend, and the few did it, and it may be questioned whether, even with the aid of antiseptics, the result is any more satisfactory.

The fathers are falling by the way. Let us each and all treasure their work, be inspirited by their devotion to the highest good irrespective of personal considerations, and so make the memory of their valuable productions the touch-stone of our own aspirations, our own ambitions.

